# Escaped

**Published:** 1905-08

**Authors:** 


Escaped.—The *Cleveland Medical Journal* says that 535 members resigned from the American Medical Association last year, and adds: "It is interesting to know that this number felt that the Association meant so little to them that they could afford to resign."

Was *that* the reason? or were they disgusted with bossism and trades union methods?

